# Unifying Terrain Awareness for the Visually Impaired through Real-Time Semantic Segmentation

**DOI:** 10.3390/s18051506

**Published:** 2018-05-10

**Authors:** Kailun Yang, Kaiwei Wang, Luis M. Bergasa, Eduardo Romera, Weijian Hu, Dongming Sun, Junwei Sun, Ruiqi Cheng, Tianxue Chen, Elena López

**Affiliations:** 1 State Key Laboratory of Modern Optical Instrumentation, Zhejiang University, Hangzhou 310027, China; elnino@zju.edu.cn (K.Y.); huweijian@zju.edu.cn (W.H.); rickycheng@zju.edu.cn (R.C.); 2 Department of Electronics, University of Alcalá, Madrid 28805, Spain; luism.bergasa@uah.es (L.M.B.); eduardo.romera@edu.uah.es (E.R.); elena.lopezg@uah.es (E.L.); 3 Department of Computing, Imperial College London, London SW7 2AZ, UK; dongming.sun17@imperial.ac.uk; 4 KR-VISION Technology Co., Ltd., Hangzhou 310023, China; junwei.sun@krvision.cn; 5 Department of Electrical and Computer Engineering, University of California, Los Angeles, CA 90095, USA; tianxuechen@ucla.edu

**Keywords:** navigation assistance, semantic segmentation, traversability awareness, obstacle avoidance, RGB-D sensor, visually-impaired people

## Abstract

Navigational assistance aims to help visually-impaired people to ambulate the environment safely and independently. This topic becomes challenging as it requires detecting a wide variety of scenes to provide higher level assistive awareness. Vision-based technologies with monocular detectors or depth sensors have sprung up within several years of research. These separate approaches have achieved remarkable results with relatively low processing time and have improved the mobility of impaired people to a large extent. However, running all detectors jointly increases the latency and burdens the computational resources. In this paper, we put forward seizing pixel-wise semantic segmentation to cover navigation-related perception needs in a unified way. This is critical not only for the terrain awareness regarding traversable areas, sidewalks, stairs and water hazards, but also for the avoidance of short-range obstacles, fast-approaching pedestrians and vehicles. The core of our unification proposal is a deep architecture, aimed at attaining efficient semantic understanding. We have integrated the approach in a wearable navigation system by incorporating robust depth segmentation. A comprehensive set of experiments prove the qualified accuracy over state-of-the-art methods while maintaining real-time speed. We also present a closed-loop field test involving real visually-impaired users, demonstrating the effectivity and versatility of the assistive framework.

## 1. Introduction

In this paper, the main purpose is focused on navigation assistance for visually-impaired people in terrain awareness, a technical term that was originally coined for commercial aircraft. In aviation, a Terrain Awareness and Warning System (TAWS) is generally an on-board module aimed at preventing unintentional impacts with the ground [1]. Within a different context, precisely blind assistance, the task of terrain awareness involves traversable ground parsing and navigation-related scene understanding, which are widely desired within the visually-impaired community [2,3].

According to the World Health Organization (WHO), an estimated 253 million people live with vision impairment, 36 million of whom are totally blind [4]. Over the past decade, the striking improvement of Computer Vision (CV) has been an enormous benefit for the Visually-Impaired (VI), allowing individuals with blindness or visual impairments to access, understand and explore surrounding environments [3,5,6]. These trends have accelerated the proliferation of monocular detectors and cost-effective RGB-Depth (RGB-D) sensors [5], supposing essential prerequisites to aid perception and navigation in visually-impaired individuals by leveraging robotic vision [7]. Along this line, a broad variety of navigational assistive technologies have been developed to accomplish specific goals including avoiding obstacles [8,9,10,11,12,13,14,15,16,17], finding paths [18,19,20,21,22,23,24,25,26,27,28,29], locating sidewalks [30,31,32,33], ascending stairs [34,35,36,37,38] or descending steps [39,40] and negotiating water hazards [41].

As a matter of fact, each one of these navigational tasks has been well tackled through its respective solutions, and the mobility of the visually impaired has been enhanced. Along with the increasing demand during everyday independent navigation [2,3], the assistance topic highlights challenges in juggling multiple tasks simultaneously and coordinating all of the perception needs efficiently. In response to these observations, the research community has been motivated to offer more independence by integrating different detectors at the basis of traversability perception, which is considered as the backbone of any VI-dedicated navigational assistive tool [26].

However, a majority of processing pursues a sequential pipeline instead of a unified way, separately detecting different navigation-related scene elements. Thereby, it is computationally intensive to run multiple detectors together, and the processing latency makes it infeasible within the blind assistance context. For illustration, one of the pioneering works [23,35,38] performed two main tasks for its personal guidance system. It approximately runs the full floor segmentation at 0.3 Frames Per Second (FPS) with additional stair detection iteration time ranging from 50–150 ms [35]. In spite of being precise in staircase modeling, this approach depends on further optimization to provide assistance at normal walking speed. A more recent example could be the sound of vision system [16,17,29], which aims to support impaired people to autonomously navigate in complex environments. While their fusion-based imaging was visually appealing, a long latency was incurred when identifying the elements of interest such as ground, walls and stairs. It takes more than 300 ms to compute stereo correspondences and detect negative obstacles [17], let alone other processing components that make it non-ideal for real-time assistance on embedded platforms. This system should be enhanced by avoiding significant delays in its main processing pipeline. Towards this objective, multi-threading is an effective way to reduce latency while sharing computational burden between cores. The commercial version of smart glasses from KR-VISION [42] has shown satisfactory performance for the detection of obstacles and hazardous curbs across different processing threads. It continuously receives images from the sensors and multi-tasks at different frame rates. Alternatively, a unified feedback design was proposed to complement the discrete detection of traversable areas and water puddles within a polarized RGB-Depth (pRGB-D) framework [41]. However, the user study revealed a higher demand for discerning terrain information.

In the literature, a number of systems [43,44,45,46] rely on sensor fusion to understand more of the surrounding scenes. Along this line, proof-of-concepts were also investigated in [47,48,49,50] to use highly integrated radars to warn against collisions with pedestrians and cars, taking into consideration that fast-moving objects are response-time critical. Arguably, for navigation assistance, an even greater concern lies in the depth data from almost all commercial 3D sensors, which suffer from a limited depth range and could not maintain the robustness across various environments [22,26,29,37]. Inevitably, approaches based on a stereo camera or light-coding RGB-D sensor generally perform range expansion [13,14], depth enhancement [22] or depend on both visual and depth information to complement each other [23]. Not to mention the time consumption in these steps, underlying assumptions were frequently made such as: the ground plane is the biggest area [9,10]; the area directly in front of the user is accessible [18,19]; and variant versions of flat world [24,36], Manhattan world [23,27,35,38] or stixel world assumptions [15,25,41]. These factors all limit the flexibility and applicability of navigational assistive technologies.

**Figure 1:**
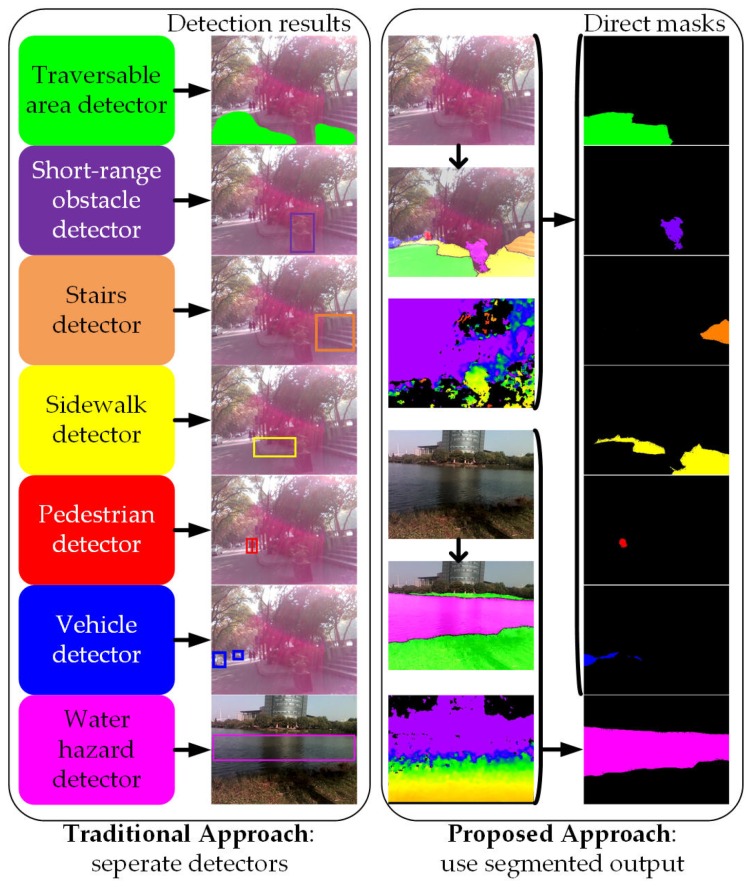
Two approaches of perception in navigational assistance for the visually impaired. A different example image is used for water hazards’ detection, but these images are all captured in real-world scenarios and segmented with the proposed approach.

Nowadays, unlike the traditional approaches mentioned above, Convolutional Neural Networks (CNNs) learn and discriminate between different features directly from the input data using a deeper abstraction of representation layers [51]. More precisely, recent advances in deep learning have achieved break-through results in most vision-based tasks including object classification [52], object detection [53], semantic segmentation [54] and instance segmentation [55]. Semantic segmentation, as one of the challenging tasks, aims to partition an image into several coherent semantically-meaningful parts. As depicted in Figure 1, because traditional approaches detect different targets independently [56], assistive feedback to the users are generated separately. Intuitively, it is beneficial to cover the tasks of the perception module of a VI-dedicated navigational assistive system in a unified manner, because it allows solving many problems at once and exploiting their inter-relations and spatial-relationships (contexts), creating reasonably favorable conditions for unified feedback design. Semantic segmentation is meant to fulfill exactly this purpose. It classifies a wide spectrum of scene classes directly, leading to pixel-wise understanding, which supposes a very rich source of processed information for upper-level navigational assistance in visually-impaired individuals. Additionally, the incessant increase of large-scale scene parsing datasets [57,58,59] and affordable computational resources has also contributed to the momentum of CNN-based semantic segmentation in its growth as the key enabler, to cover navigation-related perception tasks [56].

Based on these notions, we propose to seize pixel-wise semantic segmentation to provide terrain awareness in a unified way. Up until very recently, pixel-wise semantic segmentation was not usable in terms of speed. To respond to the surge in demand, efficient semantic segmentation has been a heavily researched topic over the past two years, spanning a diverse range of application domains with the emergence of architectures that could reach near real-time segmentation [60,61,62,63,64,65,66,67,68]. These advances have made possible the utilization of full scene segmentation in time-critical cases like blind assistance. However, to the best of our knowledge, approaches that have customized real-time semantic segmentation to assist visually-impaired pedestrians are scarce in the state of the art. In this regard, our unified framework is a pioneering attempt going much further than simply identifying the most traversable direction [28,41], and it is different from those efforts made to aid navigation in prosthetic vision [27,69,70] because our approach can be used and accessed by both blind and partially-sighted individuals.

We have already presented some preliminary studies related to our approaches [22,41]. This paper considerably extends previously-established proofs-of-concept by including novel contributions and results that reside in the following main aspects:

- A unification of terrain awareness regarding traversable areas, obstacles, sidewalks, stairs, water hazards, pedestrians and vehicles.
- A real-time semantic segmentation network to learn both global scene contexts and local textures without imposing any assumptions, while reaching higher performance than traditional approaches.
- A real-world navigational assistance framework on a wearable prototype for visually-impaired individuals.
- A comprehensive set of experiments on a large-scale public dataset, as well as an egocentric dataset captured with the assistive prototype. The real-world egocentric dataset can be accessed at [71].
- A closed-loop field test involving real visually-impaired users, which validates the effectivity and the versatility of our solution, as well as giving insightful hints about how to reach higher level safety and offer more independence to the users.

The remainder of this paper is structured as follows. Section 2 reviews related work that has addressed both traversability-related terrain awareness and real-time semantic segmentation. In Section 3, the framework is elaborated in terms of the wearable navigation assistance system, the semantic segmentation architecture and the implementation details. In Section 4, the approach is evaluated and discussed regarding real-time/real-world performance by comparing to traditional algorithms and state-of-the-art networks. In Section 5, a closed-loop field test is fully described with the aim to validate the effectivity and versatility of our approach. Section 6 draws the conclusions and gives an outlook to future work.

## 2. Related Work

In this section, we review the relevant literature on traversability/terrain awareness and pixel-wise semantic segmentation for the visually impaired.

### 2.1. Traversability Awareness

The study of the traversable part of a surface is usually referred to as traversability [22,41], which has gained huge interest within the research community of blind assistance. Among the literature, a large part of the proposals detected traversability with a commercial stereo camera. As one of the most representative, RANdom SAmpling Consensus (RANSAC) [72] is adapted to model the ground plane. A. Rodríguez et al. [9,10] estimated the ground plane based on RANSAC and filtering techniques by using the dense disparity map. Multiple variations of the RANSAC approach were reported later, each trying to improve the classic approach [12,22]. Furthermore, ground geometry assessment [29] and surface discontinuity negotiation [73] were addressed, taking into account that real-world ground areas are not always planar surfaces [74] and the wearable camera lenses share a distortion given the wide field of view. Inspired exactly by this observation, the stixel world [75] marked a significant milestone for flexibly representing traffic environments including the free road space, as well as static/moving obstacles. In this line, possibilities were explored to leverage the stixel-based techniques for autonomous vehicles and transfer them into assistive technology for the visually impaired [15,25,41]. To overcome the limitation of incompatible assumptions across application domains, [25] followed the Manhattan world stereo method [76] to obtain ground-to-image transformation; [15] clustered the normal vectors in the lower half of the field of view; while [41] integrated Inertial Measurement Unit (IMU) observations along with vision inputs in a straightforward way.

Another cluster of classic methods involves light-coding sensors, which are able to deliver dense depth information in indoor environments. R. Cheng et al. [20] detected ground and obstacles based on the algorithm of seeded growth within depth images. However, as the depth range of the light-coding sensor is limited, namely 0.8–4 m without direct sunshine, speckle-based approaches are just proof-of-concepts or only feasible in indoor environments. Since close-range depth imaging is desirable for safety-critical obstacle avoidance, heuristic approaches were developed to decrease the minimum range of the light-coding sensor in [13,14] by combining active speckle projecting with passive Infrared (IR) stereo matching. As far as longer traversability is regarded, A. Aladren et al. [23] robustly expanded range-based indoor floor segmentation with image intensities, pursuing a complex pipeline, which fails to provide real-time assistance. With the same concern on scene interpretation in the distance, a dual-field sensing scheme [21] was proposed by integrating a laser scanner and a camera. It interpreted far-field image data based on the appearance and spatial cues, which were modeled using the near-field interpreted data. In our previous work [22], large-scale IR stereo matching [14,77] and RGB guided filtering [78] were incorporated to enhance the multi-modal RGB-Infrared-Depth (RGB-IR-D) sensory awareness. It achieves superior detection results of the traversable area, which covers a broader field of view and a longer navigable depth range. However, what remains practically unexplored is the unified awareness of not only traversable areas, but also other navigation-related terrain classes such as stairs and water hazards.

### 2.2. Terrain Awareness

Motivated by the enhanced mobility and higher level demand of visually-impaired people, the research community has begun to integrate different terrain detectors beyond traversability awareness. In this line, the upper-level knowledge is offered by perception frameworks of stairs and curbs, which represent hazardous situations in everyday indoor and outdoor environments. T. Schwarze and Z. Zhong [36] propagated the valid ground plane measurements and tracked the stairs with a helmet-mounted egocentric stereo camera. J.J. Guerrero et al. [35,38] created a chest-mounted personal guidance system to detect ground areas and parametrize stairs in a sequential way. For descending steps’ classification, C. Stahlschmidt et al. [39] simplified the ground plane detection and considered depth jumps as the main characteristics by using the point cloud from a Time-of-Flight (ToF) sensor.

Intersection navigation is also one of the major ingredients of independent living. M. Poggi et al. [79] projected the point cloud from a top-view perspective thanks to the robust RANSAC-based ground segmentation and detected crosswalks by leveraging 3D data provided by a customized RGB-D camera and a Convolutional Neural Network (CNN). Taking steps further than the seeded growing ground/obstacle perception [20], R. Cheng et al. [80,81] proposed the real-time zebra crosswalk and crossing light detection algorithms to assist vulnerable visually-impaired pedestrians, which exhibited high robustness at challenging metropolitan intersections. In a previous work [41], we addressed water puddles’ detection beyond traversability with a pRGB-D sensor and generated stereo sound feedback to guide the visually-impaired to follow the prioritized direction for hazard avoidance. In spite of the impressive strides towards higher mobility of visually-impaired people, the detection of different terrain classes pursues a sequential manner instead of a unified way. As a consequence, it is not computationally efficient to run different detectors together, and the processing latency is deemed infeasible for time-critical blind assistance.

### 2.3. Semantic Segmentation for the Visually Impaired

Pixel-wise semantic segmentation has emerged as an extremely powerful approach to detect and identify multiple classes of scenes/objects simultaneously. However, the research topic of designing pixel-wise semantic segmentation to assist the visually impaired has not been widely investigated. A team of researchers proposed the semantic paintbrush [82], which is an augmented reality system based on a purely passive RGB-Infrared (RGB-IR) stereo setup, along with a laser pointer allowing the user to draw directly onto its 3D reconstruction. Unlike typical assistive systems, it places the user “in the loop” to exhaustively segment semantics of interest. L. Horne et al. [69,70] presented a computer system to aid in obstacle avoidance and distant object localization by using semantic labeling techniques. Although related, the produced stimulation pattern can be thought of as a low resolution, low dynamic range, distorted image, which is insufficient for our task. With similar purposes for prosthetic vision, A. Perez-Yus et al. [27] adopted a head-mounted RGB-D camera to detect free space, obstacles and scene direction in front of the user.

The Fully-Convolutional Network (FCN) [54], as the pioneering architecture for semantic segmentation, has been leveraged to detect the navigational path in [26], inherently alleviating the need for hand-crafting specific features, as well as providing a reliable generalization capability. A different piece of related work [28] has been recently presented to identify the most walkable direction for outdoor navigation, while semantic segmentation constitutes an intermediate step, followed by a spatial-temporal graph for decision making. It achieved decent accuracy for predicting a safe direction, namely 84% at a predetermined safety radius of 100 pixels. While inspiring, this work focused on the tracking of a safe-to-follow object by providing only sparse bounding-box semantic predictions and hence cannot be directly used for upper-level reasoning tasks. Similar bounding-box interpretation was addressed when ultrasonic sensors and computer vision joined forces [44] by semantically assigning a relative degree of danger, which is limited to only four categories of detected obstructions. Although sporadic efforts have been made along this line, these approaches are unable to run in real time, which is a critical issue for blind assistance. Additionally, they did not provide unified terrain awareness nor demonstrate closed-loop field navigation. Considering these reasons, this task represents a challenging and so far largely unexplored research topic.

### 2.4. Real-Time Pixel-Wise Semantic Segmentation

Semantic segmentation has been fueled by the recently emerging deep learning pipelines and architectures. Among the literature, a vital part of networks is predominantly based on FCNs [54], which were proposed to adapt CNNs, initially designed for classification, to produce pixel-wise classification outputs by making them fully convolutional. SegNet [60] is known as another revolutionary deep CNN architecture with a topologically symmetrical encoder-decoder design. Instead of storing all feature maps, SegNet uses max-pooling indexes obtained from the encoder to up-sample the corresponding feature maps for the decoder, which dramatically reduces the memory and computational cost. ENet [61] was proposed as an efficient alternative to enable the implementation of semantic segmentation in real time. Adopting views from ResNet [83], ENet was constructed with multiple bottleneck modules, which can be used for either down-sampling or up-sampling images. Unlike SegNet’s symmetric architecture, ENet has a larger encoder than its decoder as it is believed that the initial network layers should not directly contribute to classification. Instead, the encoder should rather act as good feature extractors and only pre-process the input for later portions of the network, while the decoder is only required to fine-tune the details. This simplified structure allows ENet to perform fast semantic segmentation. However, ENet sacrifices a good deal of accuracy earned by more complex architectures in order to remain efficient.

In our previous work, we proposed ERFNet [64,65], which aimed at maximizing the trade-off between accuracy/efficiency and making CNN-based segmentation suitable for applications on current embedded hardware platforms. With a similar purpose, SQNet [63] used parallel dilated convolutions and fused them as an element-wise sum to combine low-level knowledge from lower layers of the encoder, which helped with classifying the contours of objects more exactly. LinkNet [67] made an attempt to get accurate instance-level prediction without compromising processing time by linking the encoder and the corresponding decoder. These architectures have surpassed ENet in terms of pixel-exact classification of small features. For large-scale scene parsing tasks, PSPNet [84] was proposed to use a decoder with max-pooling layers with diverse widths in order to gather diverse levels of context in the last layers. However, PSPNet requires excessively large processing time, namely more than one second to predict a 2048 × 1024 high-solution image on one Nvidia TitanX GPU card. ICNet [66] proposed a compressed-PSPNet-based image cascade network that incorporates multi-resolution branches under proper label guidance. Although these networks claimed to yield near real-time inference, most of them are designed for autonomous vehicles [63,64,65], biomedical image segmentation [62] or human body part segmentation [68]. None of the current real-time segmentation approaches have been tailored for blind assistance, which is a time-critical, context-critical and safety-critical topic. In addition, architectures in the state-of-the-art have not been thoroughly tested in the real world. Based on this notion, we aim to customize real-time semantic segmentation to aid navigation in visually-impaired individuals and offer an in-depth evaluation, focusing on a quantitative analysis of real-world performance, followed by qualitative results, as well as discussions.

## 3. Approach

**Figure 2:**
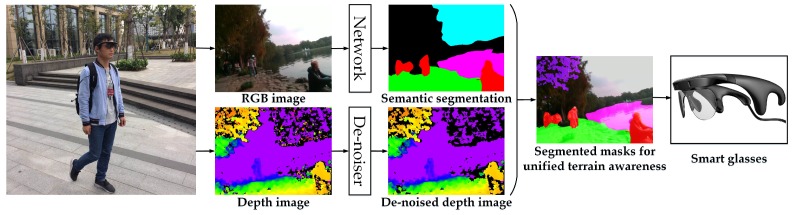
Overview of the navigation assistance system.

In this section, our approach to unify the navigation-related terrain awareness is described in detail. As shown in Figure 2, our approach is outlined in terms of the wearable navigation assistance system, which incorporates the robust depth segmentation and the real-time semantic segmentation.

### 3.1. Wearable Navigation Assistance System

#### 3.1.1. System Overview

In this framework, the main motivation is to design a prototype that should be wearable without hurting the self-esteem of visually-impaired people. With this target in mind, we follow the trend of using head-mounted glasses [22,36,41,46] to acquire environment information and interact with visually-impaired people. As worn by the user in Figure 2, the system is composed of a pair of smart glasses and a portable processor, which can be easily carried, and it is robust enough to operate in rough terrain. The pair of smart glasses, named Intoer, has been made available at [42]. Intoer is comprised of a RGB-D sensor, RealSense R200 [85], and a set of bone-conducting earphones [86].

#### 3.1.2. RGB-D Perception

**Figure 3:**
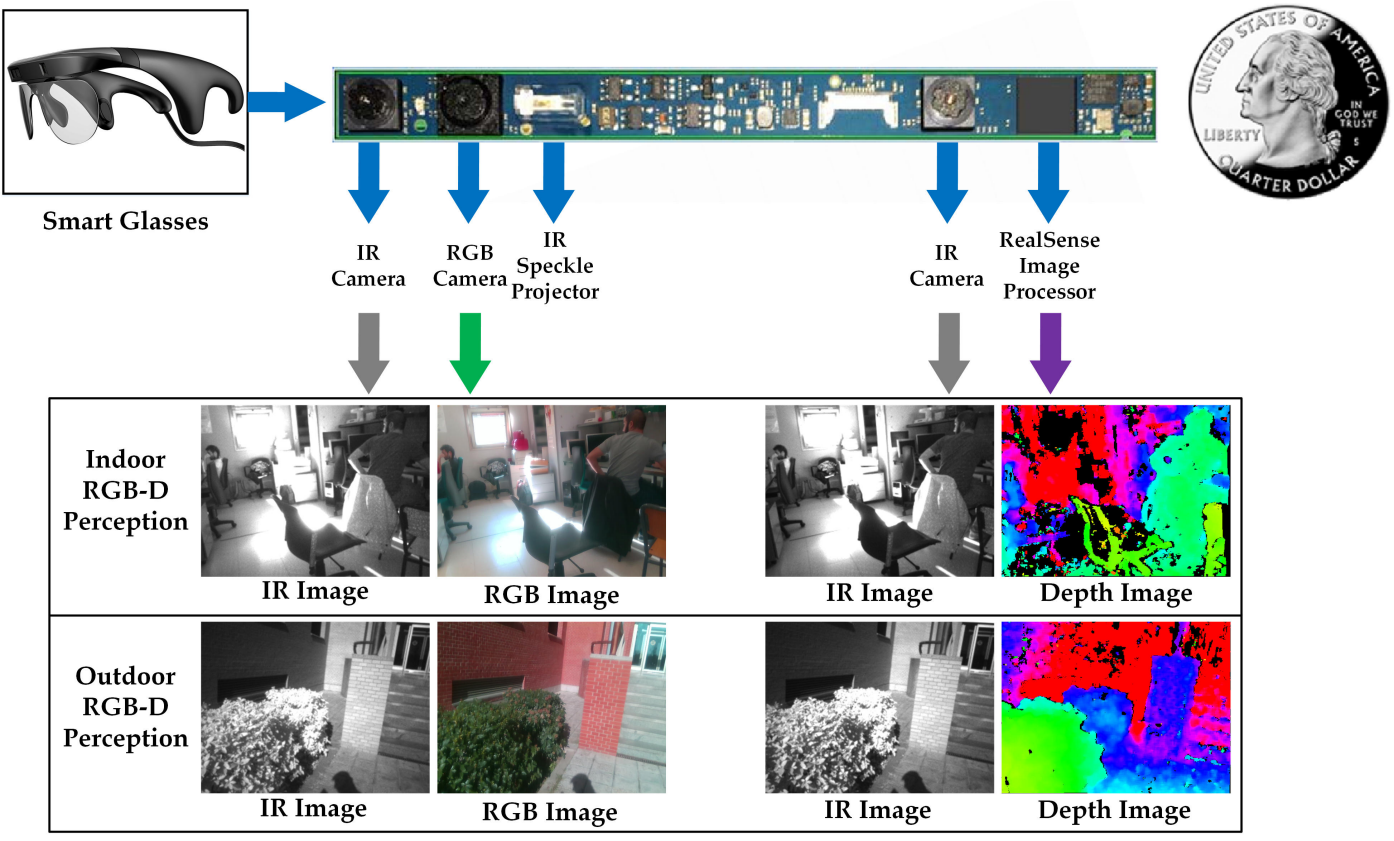
RGB-D perception of the smart glasses in indoor and outdoor environments.

Illustrated in Figure 3, this pair of smart glasses is quite suitable for navigational assistance due to its small size and light weight, as well as the environmental adaptability. Precisely, it is able to perform large-scale RGB-IR-D perception in both indoor and outdoor environments owing to the active stereo design [87]. It leverages a combination of active speckle projecting and passive stereo matching. An IR laser projector projects static non-visible near-IR patterns on the scene, which are then acquired by the left and right IR cameras. The image processor generates a depth map through the embedded stereo matching algorithm. For texture-less indoor environments, the projected patterns enrich textures. As shown in the indoor scenario in Figure 3, the texture-less black shirt hanging on the chair has been projected with plentiful near-IR patterns, which are beneficial for stereo matching to generate dense depth information. In sunny outdoor environments, shown in the outdoor scenarios in Figure 3 (see the shadow of the user on the ground), although projected patterns are submerged by sunlight, the near-IR components of sunlight shine on the scene to form well-textured IR images. With the contribution of abundant textures to robust stereo matching, the combination allows the smart glasses to work under both indoor and outdoor circumstances.

**Figure 4:**
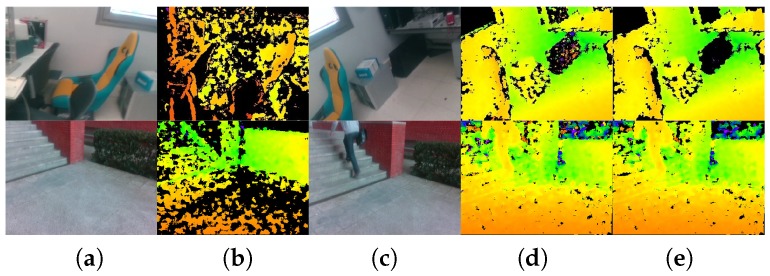
(**a**,**b**) Original RGB-D perception, depth images are sparse with many mismatched pixels; (**c**,**d**) RGB-D perception with a dense preset configuration; (**e**) depth images with noise reduction.

It is important to remark that although the pair of smart glasses enables indoor/outdoor RGB-D perception, there exist various noise sources, mismatched pixels and black holes in the depth images, as displayed in Figure 4b. According to the technique overview [85], the original depth points generated by the hardware correlation engines are high-quality photometric matches between the left-right stereo image pairs. This allows the embedded algorithm to scale well to noisy infrared images across indoor/outdoor scenarios, delivering accurate, but sparse depth information. For this reason, a large portion of pixels remain mismatched with relatively lower correlation confidence, causing many holes in the original depth image. However, in this paper, the depth image is used for robust obstacle avoidance at the basis of navigation-related terrain segmentation of CNNs. To this end, a dense depth image is preferred to assist the visually impaired so as not to leave out potential obstacles, based on the knowledge that the stereo sensor generally requires a good trade-off between density and accuracy. Following the rationale, unlike our previous work, which performed time-consuming guided hole-filing [22], we use a simple, yet effective way to deal with the noises and pre-process the depth image.

1. We enable a stream of a 640 × 480 RGB image, a stream of a 320 × 240 IR stereo pair with global shutter, which produces a high-speed stream of a 320 × 240 depth image. Depth information is projected to the RGB image so as to acquire a synchronized 640 × 480 depth stream.
2. To achieve high environmental adaptability, the automatic exposure and gain control of the IR stereo pair are enabled, while the power of the IR projector is fixed.
3. To enforce the embedded stereo matching algorithm to deliver dense maps, we use a different preset configuration with respect to the original depth image of RealSense (see Figure 4b), by controlling how aggressive the algorithm is at discarding matched pixels. Precisely, most of the depth control thresholds are at the loosest setting, while only the left-right consistency constraint is adjusted to 30 from the range [0, 2047].
4. As shown in Figure 2 and Figure 4d,e, the depth images are de-noised by eliminating small segments. Depth noises can be denoted as outliers in disparity images due to low texture, reflections, noise, etc. [88]. These outliers usually show up as small patches of disparity that is very different from the surrounding disparities. To identify these outliers, the disparity image is segmented by allowing neighboring disparities within one segment to vary by one pixel, considering a four-connected image grid. The disparities of all segments below a certain size are set to invalid. Following [77], we remove small segments with an area smaller than 200 pixels.

The dense depth image with noise reduction leads to robust segmentation of short-range obstacles when using the semantic segmentation output as the base for upper-level assistance. For illustrative purposes, in Figure 1 and Figure 2, 5 m is set as the threshold to segment directly at the pixel level if not classified as navigation-related classes including traversable area, stair, water, pedestrian or vehicle.

#### 3.1.3. Feedback Device

As far as the feedback is concerned, the bone conduction headphones transfer the detection results to the visually impaired for both terrain awareness and collision avoidance. This is important as visually-impaired people need to continue hearing environmental sounds, and the bone conducting interface allows them to hear a layer of augmented acoustic reality that is superimposed on the environmental sounds. The detailed acoustic feedback design will be introduced in Section 5.2.

### 3.2. Real-Time Semantic Segmentation Architecture

**Figure 5:**
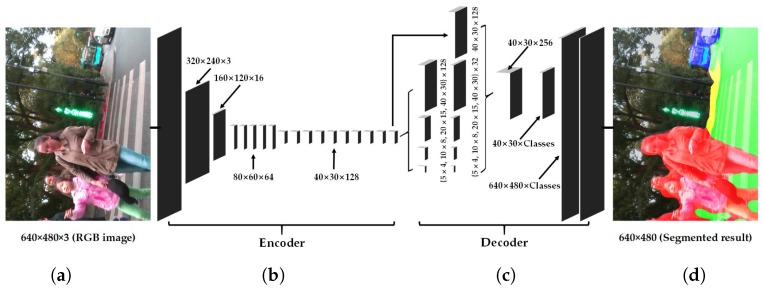
The proposed architecture. From left to right: (**a**) input, (**b**) encoder, (**c**) decoder and (**d**) prediction.

Based on the above analysis that navigation assistance is a time-critical, safety-critical, context-critical task, we design our semantic segmentation network with the corresponding key ideas shaping our approach to this project. In order to leverage the success of segmenting a variety of scenes and maintaining the efficiency, our architecture follows the encoder-decoder architecture like SegNet [60], ENet [61] and our previous ERFNet [65]. In architectures like FCN [54], feature maps from different layers need to be fused to generate a fine-grained output. As indicated in Figure 5, our approach contrarily uses a more sequential architecture based on an encoder producing down-sampled feature maps and a subsequent decoder that up-samples the feature maps to match input resolution.

#### 3.2.1. Encoder Architecture

**Figure 6:**
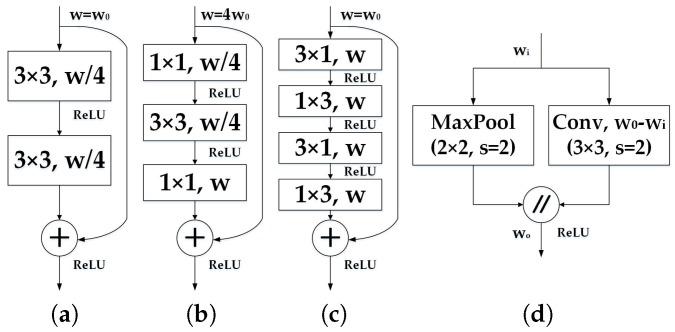
Depiction of the residual layers and the down-sampler block: (**a**) non-bottleneck, (**b**) bottleneck, (**c**) the proposed non-bottleneck-1D and (**d**) down-sampler block. *w*: width of the convolution, representing the number of feature maps, *s*: stride.

In the perspective of time-critical applications, our encoder builds upon an efficient redesign of convolutional blocks with residual connections. Residual connections [83] were supposed a breakthrough because the degradation problem could be avoided, which is present in architectures with a large amount of stacked layers. Residual layers have the property of allowing convolution layers to approximate residual functions. Formally, the output *y* of a layer vector input *x* becomes:

(1) $$y=F\left(x,W_{i}\right)+W_{s}x$$

where $W_{s}$ is usually an identity mapping and $F(x,W_{i})$ represents the residual mapping to be learned. This residual formulation facilitates learning and significantly reduces the degradation problem present in architectures that stack a large amount of layers [83]. The original work proposes two instances of this residual layer: the non-bottleneck design with two 3 × 3 convolutions as depicted in Figure 6a, or the bottleneck version as depicted in Figure 6b. Both versions have a similar number of parameters and enabled almost equivalent accuracy. However, the bottleneck requires less computational resources, and these scale in a more economical way as depth increases. For this reason, the bottleneck design has been commonly adopted in state-of-the-art networks [61,83]. However, it has been reported that non-bottleneck ResNets gain more accuracy from increased depth than the bottleneck versions, which indicates that they are not entirely equivalent, and the bottleneck design still suffers from the degradation problem [83,89].

It is worthwhile to review the redesign of the non-bottleneck residual module in our previous work [64]. As demonstrated in [90], any 2D filter can be represented by a combination of 1D filters in the following way. Let $W\in R^{C\times d^{h}\times d^{v}\times F}$ denote the weights of a typical 2D convolutional layer, where *C* is the number of input planes, *F* is the number of output planes (feature maps) and $d^{h}\times d^{v}$ represents the kernel size of each feature map (typically $d^{h}\equiv d^{v}\equiv d$). Let $b\in R^{F}$ be the vector representing the bias term for each filter and **f${}^{i}$**$\in R^{d^{h}\times d^{v}}$ represent the *i*-th kernel in the layer. Common approaches first learn these filters from data and then find low-rank approximations as a post-processing step [91]. However, this approach requires additional fine tuning, and the resulting filters may not be separable. Instead, ref. [92] demonstrates that it is possible to relax the rank-1 constraint and essentially rewrite **f${}^{i}$** as a linear combination of 1D filters:

(2) $$f^{i}=\sum_{k=1}^{K}\sigma_{k}^{i}{\bar{v}}_{k}^{i}{\left({\bar{h}}_{k}^{i}\right)}^{T}$$

where ${\bar{v}}_{k}^{i}$ and ${\left({\bar{h}}_{k}^{i}\right)}^{T}$ are vectors of length *d*, $\sigma_{k}^{i}$ is a scalar weight and *K* is the rank of **f${}^{i}$**. Based on this representation, J. Alvarez and L. Petersson [90] proposed that each convolutional layer can be decomposed with 1D filters, which can additionally include a non-linearity φ(·) in between. In this way, the *i*-th output of a decomposed layer $a_{i}^{1}$ can be expressed as a function of its input $a_{*}^{0}$ according to the following manner:

(3) $$a_{i}^{1}=\varphi \left(b_{i}^{h}+\sum_{l=1}^{L}{\bar{h}}_{il}^{T}*\left[\varphi \left(b_{l}^{v}+\sum_{c=1}^{C}{\bar{v}}_{lc}*a_{c}^{0}\right)\right]\right)$$

where *L* represents the number of filters in the intermediate layer and φ(·) can be implemented with activation functions ReLU [52] or PReLU [93]. The resulting decomposed layers have intrinsically low computational cost and simplicity. Additionally, the 1D combinations improve the compactness of the model by minimizing redundancies (as the filters are shared within each 2D combinations) and theoretically improve the learning capacity by inserting a non-linearity between the 1D filters [90].

Considering an equal kernel size *d* for simplicity, it is trivial to see that the decomposition reduces $W_{2D}\in R^{C\times d\times d\times F}$ of any 2D convolution into a pair of $W_{1D}\in R^{C\times d\times F}$, resulting in the equivalent dimensions of each 1D pair in dim=2×(C×d×F). For this reason, this factorization can be leveraged to reduce the 3 × 3 convolutions on the original residual modules. While larger filters would be benefited by this decomposition, applying it on 3 × 3 convolutions already yields a 33% reduction in parameters and further increases its computational efficiency.

By leveraging this decomposition, “Non-bottleneck-1D” (Non-bt-1D) was proposed in previous work [64,65], as depicted in Figure 6c. It is a redesign of the residual layer to strike a rational balance between the efficiency of the bottleneck and the learning capacity of non-bottleneck, by using 1D factorizations of the convolutional kernels. Therefore, it enables an efficient use of a minimized amount of residual layers to extract feature maps and achieve semantic segmentation in real time.

In addition, our down-sampler block (Figure 6d) inspired by the initial block of ENet [61] performs down-sampling by concatenating the parallel outputs of a single 3 × 3 convolution with stride 2 and a max-pooling module. It is true that down-sampling has the drawback of resulting in coarser outputs, but it also has two benefits: it enables the deeper layers to gather more context, leading to better classification, and it helps to reduce computation, as well as allowing for more complex layers in the decoder. Still, we argue that for the visually impaired, contextual information is more important than pixel-exact small features. In this regard, we perform three down-samplings to maintain a judicious trade-off between learning textures and extracting contextual information. Table 1 gives a detailed description of the integral architecture, where the redesigned residual layers are stacked in the encoder after corresponding down-samplers with different dilation rates.

#### 3.2.2. Decoder Architecture

For the terrain awareness in intelligent assistance, we propose to attach a different decoder with respect to the previous work. This key modification aims to collect more contextual information while minimizing the sacrifice of learning textures. Global context information is of cardinal signification for terrain awareness in order to prevent the feedback of confusing semantics. To detail this, several common issues are worthwhile to mention for context-critical blind assistance:

- The context relationship is universal and important especially for complex scene understanding. If the network mispredicts descending stairs in front of a lake, the visually impaired would be left vulnerable in dynamic environments. The common knowledge should be learned by the data-driven approach that stairs are seldom over a lake.
- There are many class label pairs that are texture-confusing in classification such as sidewalk/pavement versus roadways. For visually-impaired people, it is desired to identify the traversable areas that are sidewalks beyond the detection of “walkable” ground planes. Following this rationale, such distinctions should be made consistently.
- Scene targets such as pedestrians and vehicles have arbitrary sizes from the sensor perspective. For close-range obstacle avoidance and long-range warning of the fast-approaching objects, a navigation assistance system should pay much attention to different sub-regions that contain inconspicuous-category stuff.

These risks could be mitigated by exploiting more context and learning more relationships between categories. With this target in mind, we reconstruct the decoder architecture. In this reconstruction, the decoder architecture follows the pyramid pooling module as introduced by PSPNet [84]. This module is applied to harvest different sub-region representations, followed by up-sampling and concatenation layers to form the final feature representation. As a result, it carries both local and global contextual information from the pooled representations at different locations. Since it fuses features under a group of different pyramid levels, the output of different levels in this pyramid pooling module contains the feature map from the encoder with varied sizes. To maintain the weight of global features, we utilize a convolution layer after each pyramid level to reduce the dimension of context representation to 1/*N* of the original one if the level size of the pyramid is *N*. As for the situation in Figure 5c, the level size *N* equals four, and we decrease the number of feature maps from 128–32. Subsequently, the low-dimension feature maps are directly up-sampled to obtain the same size features as the original feature map through bilinear interpolation. Figure 6 contains a depiction of the feature maps generated by each of the blocks in our architecture, from the RGB input to the pixel-level class probabilities and final prediction.

### 3.3. Implementation Details

#### 3.3.1. Dataset

In our work, the challenging ADE20K dataset [57] is chosen as it covers both both indoor and outdoor scenarios. Furthermore, this dataset contains traversability-related classes and many scenes that are very important for navigation assistance such as stairs and water areas. To enrich the training dataset, we add some of the images that have the classes of sky, floor, road, grass, sidewalk, ground, water and stairs from the PASCAL-Context dataset [58] and the COCO-Stuff 10K dataset [59]. Hence, the training involves 37,075 images, within which 20,210 images are from ADE20K, 8733 images from PASCAL-Context and the remaining 8132 images from COCO-Stuff. In addition, we have 2000 images from ADE20K for validation. To provide awareness regarding the scenes that visually-impaired people care the most about during navigation, we only use the most frequent 22 classes of scenes or objects for training. Additionally, we merge the water, sea, river, pool and lake into a class of water hazards. In a similar way, the stairs, stairway and staircase are merged into a class of stairs. In total, the training involves 24 classes: water areas, stairs and 22 frequent scene elements.

#### 3.3.2. Data Augmentation

To robustify the model against the various types of images from the real world, we perform a group of data augmentations. Firstly, horizontally flipping with a 50% chance, random cropping and random scaling are jointly used to resize the cropped regions into 320 × 240 input images. Secondly, a random rotation is implemented without cropping by sampling distributions from the range [−20${}^{\circ }$, 20${}^{\circ }$]. This intuition comes from the fact that during navigation, the orientation of the smart glasses would be constantly changing and the images would rotate. This is also beneficial to eliminate the needs of previously-used IMU-based processing [22,43] that requires reasonable synchronization between IMU observations and vision inputs, which partially hinders real-time feedback. Thirdly, color jittering in terms of brightness, saturation, contrast and hue is applied. Jittering factors regarding brightness, saturation and contrast here are chosen uniformly from the range [0.8, 1.2]. Hue augmentation is performed by adding a value from the range [−0.2, 0.2] to the hue value channel of the Hue Saturation Value (HSV) representation.

#### 3.3.3. Training Setup

**Figure 7:**
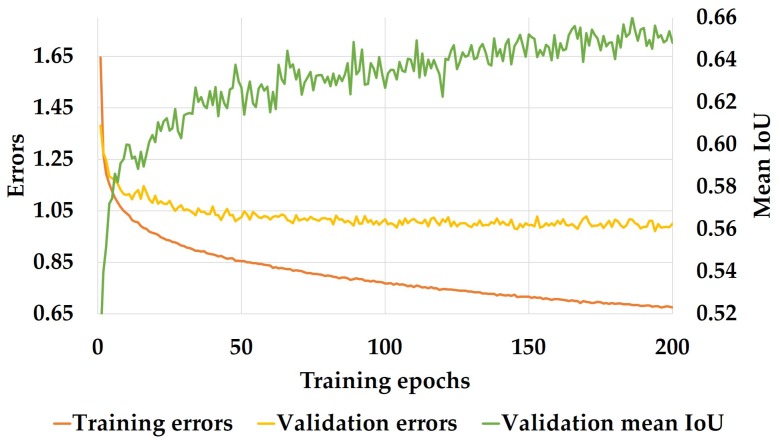
Training progress, IoU: Intersection-over-Union.

Our model is trained using the Adam optimization of stochastic gradient descent [94]. In this work, training is operated with a batch size of 12, momentum of 0.9 and weight decay of 2 × 10${}^{-4}$, and we start with an original learning rate of 5 × 10${}^{-5}$ that decreases exponentially across epochs. Following the scheme customized in [61], the weights are determined as *w*${}_{class}$ = 1/ln(*c* + *p*${}_{class}$), while *c* is set to 1.001 to enforce the model to learn more information of the less frequent classes in the dataset. For pre-training, we first adapt the encoder’s last layers to produce a single classification output by adding extra pooling layers and a fully-connected layer and finally train the modified encoder on ImageNet [95]. After that, the extra layers are removed, and the decoder is appended to train the full network. In the training phase, we also include Batch-Normalization (BN) [96] to accelerate convergence and dropout [97] as a regularization measure. More precisely, the dropout probability is set to 0.3 in the encoder and 0.1 in the decoder respectively, as this yielded better results in our architecture. With this setup, the training reaches convergence, as shown in Figure 7, when the cross-entropy loss value is used as the training criterion.

## 4. Experiments and Discussions

In this section, we performed a comprehensive set of experiments to prove the qualified accuracy and speed for navigation assistance, as well as the real-world performance by comparing with traditional algorithms and state-of-the-art networks.

### 4.1. Experiment Setup

The experiments were performed with the wearable navigation systems in public spaces around Westlake, the Zijingang Campus and the Yuquan Campus at Zhejiang University in Hangzhou and the Polytechnic School at University of Alcalá in Madrid, as well as Venice Beach and University of California in Los Angeles. When navigating in different scenarios, we captured real-world images while keeping moving by using our head-worn smart glasses available at [42]. In this fashion, a real-world egocentric vision dataset can be accessed from the TerrainAwarenessDataset [71]. The metrics reported in this paper correspond to Intersection-over-Union (IoU) and Pixel-wise Accuracy (PA), which prevail in semantic segmentation tasks [57,58]:

(4) $$IoU=\frac{TP}{TP+FP+FN}$$

where *TP*, *FP* and *FN* are respectively the number of True Positives, False Positives and False Negatives at the pixel level,

(5) $$PA=\frac{CCP}{LP}$$

where *TP* and *LP* are respectively the number of Correctly-Classified Pixels and Labeled Pixels.

### 4.2. Real-Time Performance

The total computation time of a single frame is 16 ms, while the image acquisition and preprocessing from the smart glasses take 3 ms, and the time cost for the semantic segmentation is 13 ms (at 320 × 240). In this sense, the computation cost is saved to maintain a reasonably qualified refresh-rate of 62.5 FPS on a cost-effective processor with a single GPU GTX1050Ti. This inference time demonstrates that it is able to run our approach in real time, while allowing additional time for auditory [9,22,41] or tactile feedback [11,13,43]. In this paper, we use a highly customized set of stereo sound feedback for assistive awareness. It takes around 40 ms for the sonification of the semantic masks, which will be introduced in Section 5.2. Additionally, on an embedded GPU Tegra TX1 (Jetson TX1) that enables higher portability while consuming less than 10 Watts at full load, our approach achieves approximately 22.0 FPS.

In this experiment, we compare the real-time performance of our architecture with state-of-the-art networks that are designed for efficient semantic segmentation. Table 2 displays the inference time (forward pass) for different resolutions (including 320 × 240, 448 × 256, 640 × 480) on a cost-effective GPU GTX1050Ti. At 320 × 240, a resolution that is enough to recognize any urban scene accurately for navigation assistance, our architecture is the fastest, namely 13 ms. Admittedly, the runtime of SegNet [60] and LinkNet [67] at this resolution is not able to be tested due to the inconsistent tensor sizes at down-sampling layers. For this reason, we test at 448 × 256, another efficient resolution at which most of the architectures can be evaluated. Furthermore, our model is super fast, second to LinkNet [67]. At 640 × 480, a resolution that is close to the average width/height of images from the ADE20K dataset [57], ENet [61] is the fastest, while the runtime of our model is 34 m, resulting in a 29.4 FPS of frame rate. However, for navigation assistance, 320 × 240 is arguably the optimum resolution of the three resolutions, since pixel-exact features are less desired by visually-impaired people, but require higher input resolution, as well as longer processing latency. Still, the average IoU value tested on the ADE20K dataset of our architecture is apparently higher than ENet and LinkNet. When comparing with our previous work, both the speed and accuracy of our architecture are slightly better than ERFNet [65], which was designed for autonomous driving. Our ERF-PSPNet inherits the encoder design, but implements a different decoder, which becomes quite efficient at resolutions that are suitable for navigation assistance. In summary, our network achieves a speed that is as competitively fast as the fastest ones (ENet, LinkNet), while having a significantly better accuracy.

### 4.3. Segmentation Accuracy

The accuracy of our approach is firstly evaluated on the challenging ADE20K dataset [57] by comparing the proposed ERF-PSPNet with deep neural networks in the state-of-the-art for real-time segmentation including UNet [62], SegNet [60], ENet [61], SQNet [63], LinkNet [67] and our previous ERFNet [65]. Table 3 details the accuracy of traversability-related classes including floor, road, grass, sidewalk, ground and other important navigation-related classes including sky, person, car, water and stairs. In our implementation, the IoU value of ENet is higher than SQNet and LinkNet on the ADE20K dataset, which is a challenging dataset requiring the architecture to learn rich contextual information. Since ENet applies multiple dilated convolution layers in order to take a wider context into account, it outperforms SQNet and LinkNet, even though these two networks claimed to achieve higher accuracy than ENet on the datasets for intelligent vehicles. As far as our architecture is concerned, it could be said that the accuracy of most classes obtained with the proposed ERF-PSPNet exceeds the state-of-the-art architectures that are also designed for real-time applications, especially the important improvements achieved on water and stairs. Our architecture builds upon previous work, but has the ability to collect more contextual information without the major sacrifice of learning from textures. As a result, only the accuracy values of sky and person are slightly lower than ERFNet.

### 4.4. Real-World Performance

To analyze the major concern of detection performance for real-world assistance, we collect results over several depth ranges: within 2 m, 2–3 m, 3–5 m and 5–10 m on the TerrainAwarenessDataset [71], which contains 120 images for testing with fine annotations of seven important classes for navigation assistance including: sky, ground, sidewalks, stairs, water hazards, persons and cars. This adequately considers that in navigational assistance, 2 m is the general distance for avoiding static obstacles, while the warning distance should be longer when a moving object approaches, e.g., 3 m for pedestrians and 10 m for cars in urban environments. In addition, the short range of ground area detection helps to determine the most walkable direction [28], while superior path planning could be supported by longer traversability awareness [22], e.g., 5–10 m. Table 4 shows both the IoU and pixel-wise accuracy of traversability awareness, which is the core task of navigational assistance. Here, the traversable areas involve the ground, floor, road, grass and sidewalk.

We compare the traversable area detection of our ERF-PSPNet to state-of-the-art architectures and a depth-based segmentation approach 3D-RANSAC-F [9], which estimates the ground plane based on RANSAC and filtering techniques by using the dense disparity map. As the depth information of the ground area may be noisy and missing in dynamic environments, we implemented an RGB image-guided filter [78] to fill holes before detection. In this way, the traditional 3D-RANSAC-F achieves decent accuracy ranging from 2–5 m, and it excels SegNet and ENet from 2–3 m, as the depth map within this range is quite dense thanks to the active stereo design of the smart glasses. However, 3D-RANSAC-F simply segmented the ground plane from obstacles, but has no ability to distinguish traversable areas from other semantic classes such as water areas, resulting in a low IoU on the real-world dataset, where the biggest ground plane assumption fails in a vital part of the images.

Intriguingly, although ENet exceeds SegNet/LinkNet on the ADE20K dataset, it cannot generalize well in real-world scenarios due to the limited learning capacity that hinders its usability. As a result, SegNet and LinkNet exceed ENet in terms of IoU and pixel-wise accuracy when testing on our real-world dataset. UNet is a classic convolutional network for biomedical image segmentation, which suffers even more from the model capacity because it is designed to use limited available annotated samples. Despite being efficient, it continues to struggle at delivering effective segmentation and predicting high-quality semantics. Still, the proposed ERF-PSPNet outperforms 3D-RANSAC-F and these networks in both ranges by a significant margin, due to the judicious trade-off between learning capacity and inferring efficiency achieved in our architecture. As far as terrain awareness is concerned, even if the IoU is not very high, the segmentation results are still of great use. For the visually impaired, it is preferred to know that there are stairs or there is an approaching pedestrian in some direction even if the shape is not exactly accurate. Furthermore, it is observed in Table 5 that most of the pixel-wise accuracy within different ranges is over 90%, which reveals the capacity of our approach for the unification of these detection tasks. It is noteworthy that IoU values of stairs and persons on the real-world dataset that mainly contains outdoor daytime images are apparently higher than those achieved on the ADE20K dataset. Although our dataset represents totally unseen scenarios, it mainly focuses on assistance-related urban scenes, while most persons are pedestrians and stairs are close to the user. In comparison, ADE20K features a high variability of person postures and far-away stairs. In this sense, ADE20K is more challenging than the real-world dataset in terms of these two classes.

### 4.5. Qualitative Analysis

**Figure 8:**
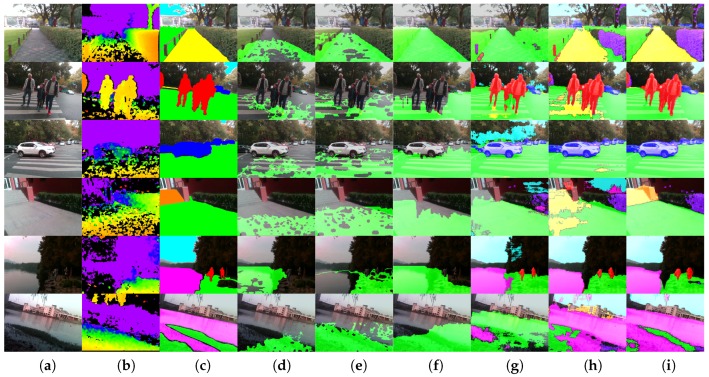
Qualitative examples of the segmentation on real-world images produced by our approach compared with ground-truth annotation, 3D-SeededRegionGrowing [20], 3D-RANSAC-F [9], FreeSpaceParse [25], ENet [61] and LinkNet [67]. From left to right: (**a**) RGB image, (**b**) depth image, (**c**) annotation, (**d**) 3D-SeededRegionGrowing, (**e**) 3D-RANSAC-F, (**f**) FreeSpaceParse, (**g**) ENet, (**h**) LinkNet and (**i**) our approach.

This subsection investigates the qualitative segmentation by comparing against traditional algorithms, as well as data-driven-based methods. Figure 8 exhibits the montage of pixel-wise results generated by our ERF-PSPNet, LinkNet [67], ENet [61], FreeSpaceParse [25], 3D-RANSAC-F [9] and 3D-SeededRegionGrowing [20].

For traditional approaches, 3D-SeededRegionGrowing and 3D-RANSAC-F both assume a plane model regarding ground area identification for visually impaired applications. This plane model can be recovered using a local approach such as 3D-SeededRegionGrowing exploring neighboring patches, or globally making use of RANSAC for ground plane equation identification. 3D-SeededRegionGrowing relies on the sensor to deliver a dense 3D point cloud and struggles at producing complete segmentation in highly-dynamic environments. Although 3D-RANSAC-F expands the detection range of the traversable area due to the global strategy, its pixel-wise parsing results are also substantially fragmented. It is worth mentioning that FreeSpaceParse [25], a procedure that renders stixel-level segmentation with the original purpose of representing traffic situations, has been applied successfully thanks to the sensor fusion [41] by utilizing attitude angles. However, the procedure tailored to the problem relies on additional IMU observations and could not differentiate between ground and water areas. This problem also exists in other traditional algorithms, while 3D-SeededRegionGrowing even completely misdetects hazardous water areas as traversable areas, due to the assumption that the ground plane should be the lowest part, as it made.

As far as the deep learning-based approaches are concerned, they have the crucial advantages of exploiting a significant amount of data, thus eliminating the dependencies on assumptions. However, for ENet and LinkNet, we can observe that sometimes, trees/walls would be misclassified as sky/ground. In addition, these networks cannot draw a distinction of ground areas vs. sidewalks consistently. This is mainly due to the incompetence to collect sufficient contextual information. Qualitatively, our approach not only yields longer and more consistent segmentation, which will definitely benefit the traversability awareness, but also retains the outstanding ability to provide the terrain awareness within this unified framework.

### 4.6. Indoor/Outdoor Detection Analysis

**Figure 9:**
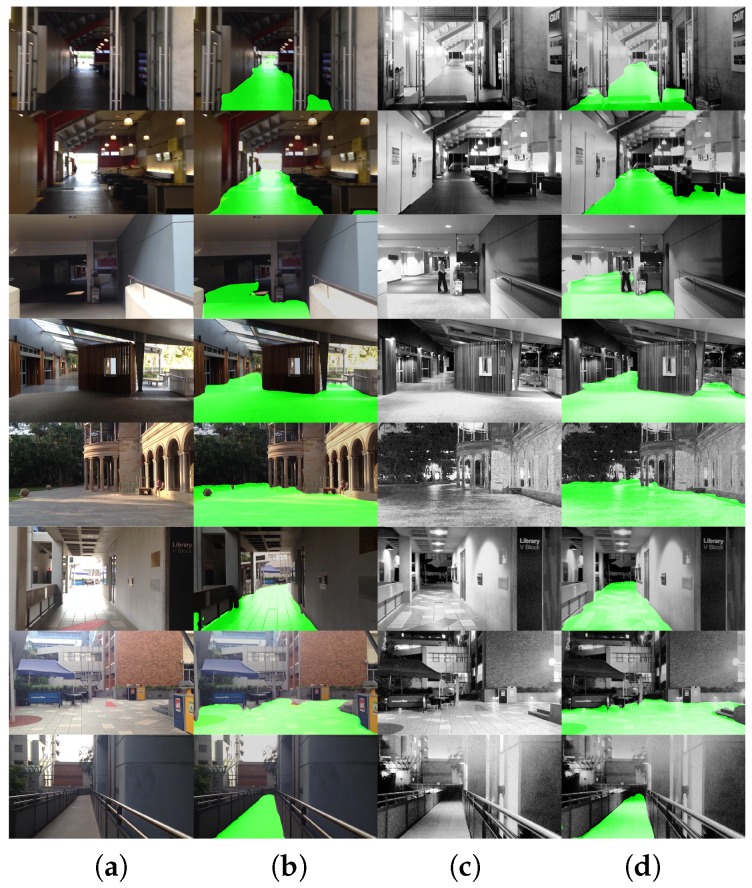
Qualitative examples of the traversable area segmentation on day/night images from the Gardens Point dataset [98] produced by our approach across indoor/outdoor environments. From left to right: (**a**) RGB images during the daytime; (**b**) traversable area parsing during the daytime; (**c**) RGB images at night; (**d**) traversable area parsing at night.

We have already proven that our sensory awareness with the smart glasses can deliver robust depth segmentation under different situations in [22]. Here, to prove that our approach can work across indoor/outdoor environments, we evaluate the traversable area segmentation of day/night scenarios from the Gardens Point dataset [98], which mainly contains ground areas in most images along the same trajectory as was originally captured for visual localization. For the reader’s information, the Gardens Point dataset was recorded while moving on the Gardens Point Campus of Queensland University of Technology in Brisbane. Qualitatively, our approach can provide quite robust and effective segmentation for traversability awareness. However, as demonstrated in Figure 9, we observe that generally in the daytime, the segmentation of outdoor scenarios is more robust than indoor cases; while at night, the indoor segmentation is slightly better than outdoors. This is mainly because most of the images we used for training are RGB images with well-balanced illumination conditions. To further enhance the robustness in the future, we aim to implement illumination-invariant image pre-transformation, as well as to incorporate near-infrared spectral and pixel-wise polarimetric information [41].

## 5. Field Test

### 5.1. Field Test Setup

**Figure 10:**
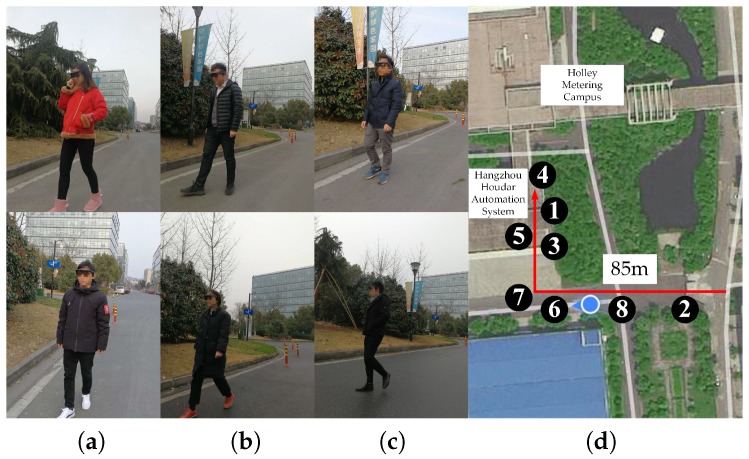
The moments of the field test. (**a**–**c**) Participants’ faces are blurred for the protection of their privacy. We have obtained approval to use the navigation assistance results for research work. (**d**) The field test around the campus with several locations marked corresponding to Figure 11.

We performed a closed-loop field test in February 2018, with six visually-impaired users around Holley Metering Campus in Hangzhou, as displayed in Figure 10. The terrain traversed involves grass, ground and pavement. After learning the stereo sound feedback of the system when wearing our smart glasses, participants had to start the navigation and reach the staircase by hearing real-time acoustic feedback. Obstacles along the trajectory (around 85 m) include low-lying traffic cones, static and moving vehicles/pedestrians, as well as other different kinds of obstacles. For safety reasons, the traffic condition is relatively peaceful compared with urban roadways, and most vehicles are at a low speed when passing through the campus.

**Figure 11:**
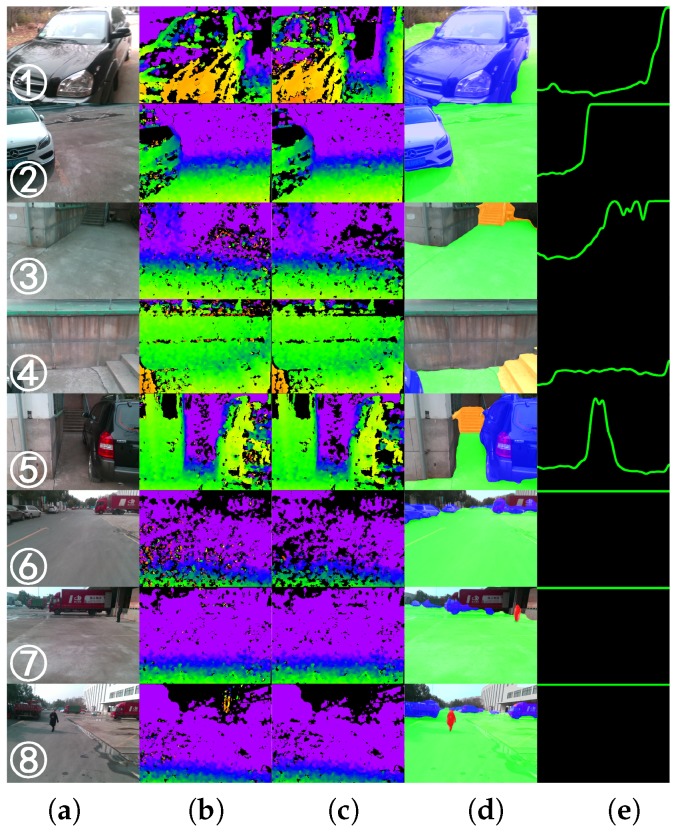
Qualitative examples of detection results and traversable lines produced by our approach in the real-world scenario of the field test, corresponding to the locations marked in Figure 10: (**a**) RGB image, (**b**) original depth image; (**c**) depth image with noise reduction; (**d**) segmentation result and (**e**) traversable line.

Figure 11 depicts typical scenarios of the field test and traversable lines, which represent the walkable distances of different directions. In each direction, the farthest distance for navigation is determined by both the traversable areas and the depth images with noise reduction. Unlike our previous work [22,41], the traversable areas are segmented using the semantic masks instead of stixel computation or depth segmentation. For illustrative purposes, the maximum traversable distance in Figure 11 is set to 9.5 m. As a result, it sometimes appears as a flat line, denoting the scenario is obstacle-free and the user should feel quite safe to walk forward. Following [41], the traversable line is mapped to the sounds of instruments, aimed to provide real-time acoustic feedback for hazard avoidance and safety awareness.

### 5.2. Stereo Sound Feedback

Inspired by the evidence that blind individuals manifest supranormal capabilities performing spatial hearing tasks [99] and high sensitivity to left/right sound signals [100], our interaction method is based on sonification, in which data are represented by sound [101]. Precisely, we use a variant of the sound-mapping interface presented in our previous work [41]. This interaction method renders real-time acoustic feedback to visually-impaired people by synthesizing stereo sound from the traversable line, which has already been proven to be efficient in navigational tasks. It aims to transfer the most walkable direction and the traversable distance of the forward direction. The most walkable way is determined using the prioritized direction to avoid close obstacles [41]. Admittedly, the indicated moving direction is partially influenced by the continuous movement of head-worn glasses during navigation. However, the IMU-based alignment is not applied because visually-impaired people usually determine the azimuth by turning the head and hearing the change of sounds. In this regard, the relative change of the sound azimuth is arguably more important than the absolute azimuth for sonification. Our approach excels at the prediction of robust semantics, which is quite suitable for the upper-level sonification of the traversable direction.

**Figure 12:**
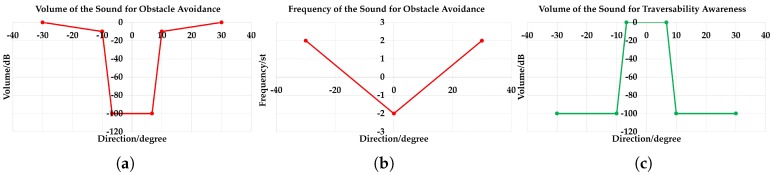
Detailed parameters of the sound mapping: (**a**) volume of the sound for obstacle avoidance; (**b**) frequency of the sound for obstacle avoidance; (**c**) volume of the sound for traversability awareness.

In this work, a clarinet sound is used as feedback for the most walkable direction, which can also be denoted as the sound for obstacle avoidance. Because the farther the user deviates from the traversable direction, the more pronounced the sound should be to warn against hazardous obstacles, as depicted in Figure 12a,b, this sound is designed to have louder volume and higher frequency at larger degrees of the stereo sound. We use the clarinet sound because it has a wide pitch range and the volume could be easily controlled within its main registers. In addition, it is suitable for continuous feedback with distinctive tone and great penetrating power. In our implementation, the volumes ranges within [−100, 0] decibels (dB), and the frequency ranges from [−2, 2] semi-tones (st), corresponding to 319–493 Hz.

When the most traversable direction indicates the forward path, a sound of traversability awareness should be fed back to the visually-impaired user. In this way, he/she would feel safe to walk without paying much attention to the sound for obstacle avoidance. Here, we use the water droplet sound for safety awareness considering one of its good properties: the sound of water droplets remains mellow when adjusting the mapping parameters. Another main advantage lies in the timbre of the clarinet and water droplet, which sound quite different, so it would not be confusing when synthesizing these two sounds simultaneously. In addition, the volume mapping is inversely related to the degree of traversable direction when compared with the sound for obstacle avoidance as revealed in Figure 12a,c. At the same time, the traversable distance of the forward path is mapped to the interval of the water droplet sound. When the walkable distance is long, the interval is short, so the user would walk briskly. When the traversable distance is limited, the interval would be relatively longer to remind the user to slow down and pay good attention to the sound for hazard awareness.

**Figure 13:**
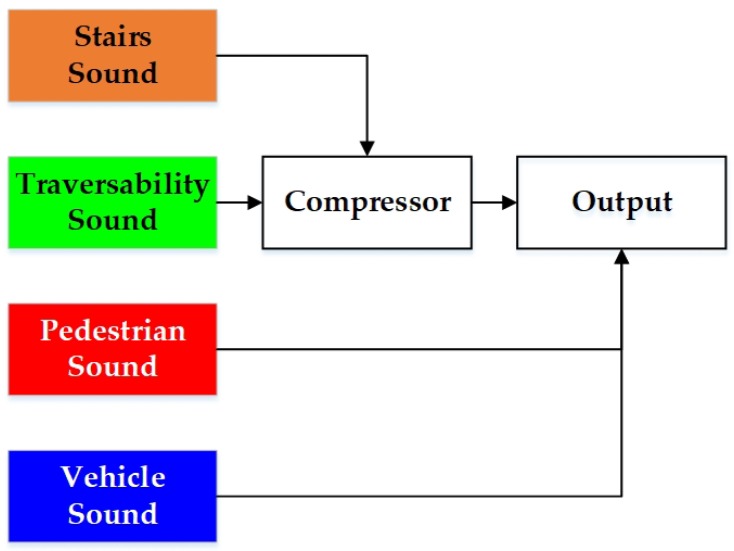
The mutual interaction between different sound mapping sources for navigation assistance.

To simplify the sonification of the semantic masks for unified terrain awareness, we use a similar approach in [9] to detect stairs, pedestrians and vehicles at the basis of semantic masks. An instance of staircase, person or car would be fed back by empirically setting 500 points as the detection threshold for 320 × 240 resolution pixel-wise segmented images within 5 m. This is also beneficial to remove false positives caused by erroneous semantic pixels due to noises. Here, the sounds of stairs, pedestrians and vehicles correspond to the instruments bell, xylophone and horn, respectively. Because a staircase represents a special traversable region, we use a compressor (see Figure 13) to reduce the volume of traversability-related feedback, including the sound for obstacle avoidance and safety awareness as introduced above. Intriguingly, the stairway sound follows the Shepard tone [102], to create the auditory illusion of a tone that continually ascends or descends in pitch, corresponding to the ascending/descending stairs. The ascending stairs and descending steps are distinguished using depth information, so visually-impaired people would perceive the terrain in advance. Our sound system is implemented with FMOD [103], which is a game sound engine supporting high-speed audio synthesis. As a result, our stereo sound feeds back the semantic information within 40 ms.

### 5.3. Field Test Results

During this assistance study, participants would learn the stereo sound feedback in the first place. The working pattern of the system and signals from the bone conduction headset were introduced. Each participant had 10 min to learn, adapt to the audio interface and wander around casually. By touching obstacles, navigating pathways and listening to the sonified sound, we had ensured that the participants fully understood the rules of the sonification-based interaction method. After that, participants were asked to navigate without collisions and reach the staircase (see the staircase images in Figure 11). To provide the participants with a sense of orientation, the users would get an extra hint to turn at the bends or the road intersection. Admittedly, in a more general usage scenario, a higher layer of knowledge could be offered by GPS (Global Positioning System) or topological localization. For readers’ information, we have also blindfolded ourselves and traversed more than 5 km without fatigue using the proposed system and AMAP [104].

In this research, all visually-impaired participants completed the test, although sometimes they had to turn to us for help as displayed in Table 6, mostly to ensure the orientation. The number of collisions and time to complete the test were also recorded. Collisions include collisions with obstacles such as traffic cones, vehicles, walls, and so on. The timer started when a participant began the navigation and stopped when the participant completed a single test. As recorded in Table 6, the number of collisions was few when traversing such a long trajectory more of than 80 m, and each user had successfully avoided at least five obstacles. In this test, explicit feedback about directions to traverse were found helpful for navigation assistance. The results suggest that participants were aware of obstacles and semantics with our system and could make use of the stereo sound to keep away from hazards including approaching pedestrians, vehicles and close-range obstacles. They had learned to navigate traversable paths and finally reached the staircase provided with the assistive awareness. In addition, most of the traversing time suggests that our system supported navigation at normal walking speed, although some individuals took a relatively longer time to finish the test. In summary, the safety and versatility of the navigation assistance system have been dramatically enhanced.

**Figure 14:**
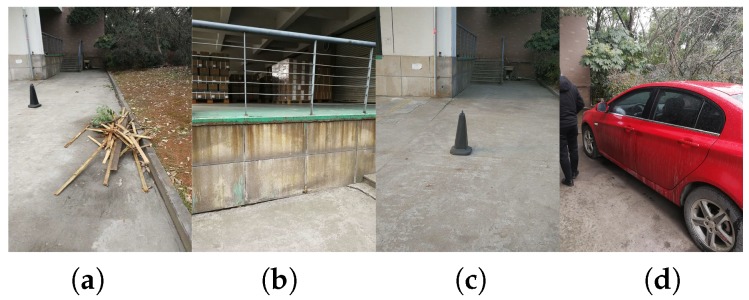
Situations of the collision with obstacles: (**a**) The wood, (**b**) The fence, (**c**) A traffic cone, (**d**) A car.

Figure 14 depicts the situations of the collisions (only four times in total). Some of the failures were due to our semantic classification framework. For example, it sometimes failed to detect low-lying obstacles as shown in Figure 14a. Instead, the wood had been misclassified as part of the ground because it represents an unseen scenario to our CNN. We believe new datasets will play an essential role to robustify the model against more unseen scenarios, even though our approach is already able to generalize far beyond its training data. Figure 14b is more related to the depth sensory awareness. When approaching the fence, the depth information would be too sparse to enable robust traversable lines. Figure 14c involves a low traffic cone on the way to the staircase. Although users could perceive it at several meters away, it would be out of the vertical field of view when getting closer. This represents a common problem of head-mounted prototypes. For Figure 14d, the collision is more related to the horizontal field of view. Such collisions occur when the user had already bypassed the obstacle, but still scratched the sides. We are interested in panoramic semantic segmentation, which would be useful to provide 360${}^{\circ }$ awareness to enhance the safety in the future.

### 5.4. Feedback Information from Participants

After the field test, the participants were asked three simple questions including whether the system gives feedback in time, whether the prototype is comfortable to wear and whether the system is useful. In this form, we have the questionnaire in Table 7, together with their suggestions for future improvement. All users answered that the system is useful and could help them to avoid obstacles and perceive semantic environmental information.

As far as the detailed feelings are concerned, User 1 thinks the warning method is acceptable, which is very helpful for obstacle avoidance. She also believes that an adaptive phase would be beneficial to better use the assistive tool. The pair of smart glasses seems to a little bit heavy, applying pressure to her nose. User 2 thinks that learning the feedback in advance helps him become comfortable with and sensitive to the sound, and the smart glasses are suitable for him to wear. User 3 thinks that the warning method is in accordance with his habits, and he hopes the assistive prototype will be more portable. User 4 thinks that the direction of the feedback is accurate and clear, even though the volume seems to be a bit on the low side. He also believes that the system would be more convenient to use if it were wireless. User 5 thinks the sound representing the traversable direction makes her feel safe, but it will be annoying in narrow passages. There is a corner (about 2 m wide) in the field test scenario. Here, sound for hazard avoidance would work due to the existence of close obstacles. For the sake of safety, the direction of the stereo sound would not be constant when the user continuously deviates from the traversable path in such situations. We believe this explains her being a bit confused at the corner (narrow passage in her mind). For the sound representing the stairs, she considers it to be very good due to the upward sensation. Although the pair of smart glasses would slide down after long-term use during her experience, she regards it as highly comfortable to wear. User 6 wants to use the prototype for more time, but he also worries that listening too much to the sound will make him agitated. Moreover, the participants are optimistic about the system and would like to have a more profound experience.

### 5.5. Maturity Analysis

**Figure 15:**
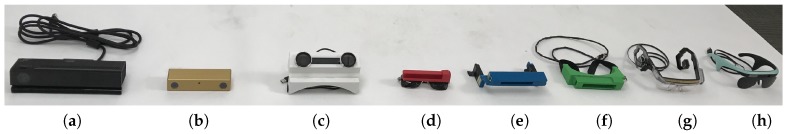
Evolution of the smart glasses: (**a**) Kinect used in [20,43], (**b**) Bumblebee stereo camera used in [9,10], (**c**) pRGB-D sensor [41], (**d**) a pair of sunglasses with sensors [13], (**e**) a 3D-printed prototype with RGB-D sensor and millimeter wave radar, (**f**) a 3D-printed prototype with RGB-D sensor and IMU [22], (**g**) a wearable navigation system [81] and (**h**) the pair of smart glasses that has been made available at [42].

Following [9,105], we offer a maturity study, along with the evolution of the smart glasses. On the one hand, our system is a prototype. It has been tested in real-world environments with visually-impaired users, which validates the approach. The prototype is designed as a pair of smart glasses, with various former designs exhibited in Figure 15. It has evolved since the original version of the hand-hold Kinect sensor (see Figure 15a), which was used in [20,43] as a proof-of-concept. It is only feasible in indoor environments due to its light-coding technology. To support outdoor usage, we have a chest-mounted prototype with a Bumblebee stereo camera (see Figure 15b), which is also similar to the one in [9,10]. In [41], the pRGB-D sensor (see Figure 15c) emerged to unify the detection of traversable directions and water puddles. However, the minimum range of the sensor is limited, posing challenges for close obstacle avoidance. Thereby, we have a pair of smart sunglasses (see Figure 15d) aimed at decreasing the minimum range of the RGB-D sensor [13]. After that, a set of low-power millimeter wave radars (see Figure 15e) were integrated to warn against fast-approaching vehicles from both sides. Figure 15f depicts the important prototype that enables adaptability across indoor/outdoor environments [22]. Figure 15g is an intermediate version targeted at intersection navigation [81]. In this paper, we discard previous invalid designs and maintain the strengths, having the mature version of smart glasses, which are easy to wear from the point of view of most users. In the near future, we would add optional accessories such as a nose pad to make the prototype more comfortable to wear for all visually-impaired people.

On the other hand, we follow the survey [8] to quantitatively evaluate the maturity. In the survey [8], 14 features and an overall score were defined as an attempt to compare the different approaches designed for visually-impaired people. Such a maturity analysis gives a measure of the system’s progress/maturity, as well as its overall satisfaction degree. In this regard, it allows us to compare our approach with traditional assistive technologies, which are not limited to vision-based solutions. Table 8 shows the average score of each feature graded by the users and developers. As the first seven features correspond to the needs of visually-impaired users, these scores were given by the participants of the field test. The remaining features reflect the views of designers, whose scores were graded by the engineers, entrepreneurs and professors.

**Figure 16:**
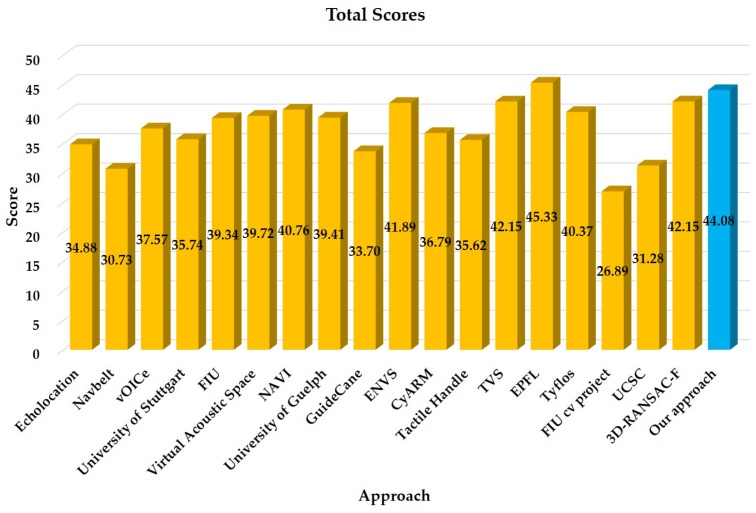
Maturity ranking, which shows the total score for each approach. From left to right: Echolocation, Navbelt, vOICe, University of Stuttgart, FIU, Virtual Acoustic Space, NAVI, University of Guelph, GuideCane, ENVS, CyARM, Tactile Handle, TVS, EPFL, Tyflos, FIU cv project, UCSC, 3D-RANSAC-F [9] and our approach.

In addition to Table 8, we also give a whole picture (see Figure 16) for the approaches including the vision-based 3D-RANSAC-F [9] rated by itself and 17 traditional obstacle avoidance systems including Echolocation, Navbelt, vOICe, a prototype designed by University of Stuttgart, FIU, Virtual Acoustic Space, NAVI, a system from University of Guelph, GuidanceCane, ENVS, CyARM, Tactile Handle, TVS, EPFL, Tyflos, FIU cv project and UCSC, which were reviewed in detail and graded by the systematic survey [8]. Among these portable obstacle detection systems, various sensors are integrated such as cameras, ultrasonic sensors and laser scanners, involving different feedback designs between auditory or tactile modalities.

In total, our proposal ranked in the top two of these approaches, second to EPFL, which achieved a score of 45.33 using a stereoscopic sonar system and vibrator-based tactile feedback, but it cannot adequately describe the 3D space and collect semantic information. Comparatively, our high score is mainly contributed by the scores on F1 (real-time), F7 (functionalities), F12 (originality) and F13 (availability). Real-time semantic segmentation allows us to provide assistive awareness in a unified way. It covers multiple perception tasks to aid navigation in visually-impaired individuals. This explains the high scores of F1 (real-time) and F7 (functionalities). As far as F12 (originality) is concerned, our approach represents one of the pioneering efforts to develop pixel-wise semantic segmentation for navigation assistance systems. F13 (availability) denotes that the system is implemented and ready for field navigation. F8 (simple) and F9 (robust) are the relative weaknesses of our framework. F8 (simple) requires the complexity of both hardware and software to be small. In this regard, deep learning-based approaches are intrinsically more complex than traditional prototypes. According to [8], F9 (robust) requires that the system still functions in the presence of partial failures. Although our approach is robust in different environmental conditions, which have been proven by the real-world experiments and the outdoor field test, the depth sensory awareness and pixel-wise semantic segmentation are coupled with each other. As far as the indoor test is concerned, we have also blindfolded ourselves to safely navigate in several buildings using our smart glasses. Because indoor environments contain less navigation-related semantics of interest, our system is more like an obstacle avoidance tool provided with superior traversability awareness, where the warning distance is closer due to safety considerations. Readers are advised to refer to our previous work [13,14,22] to learn more about the precisely-designed feedback system and relevant indoor field tests. In addition, we aim to develop semantics-aware SLAM (Simultaneous Localization And Mapping) by making full use of the indoor semantic masks such as desk, chair and door. Following the rules of the survey [8], F5 and F10 are not scored due to the usage of a single Universal Serial Bus (USB) 3.0 cord and the lack of cost information for future deployment. Despite being a possible subjective measure, it is a good reference for a numerical comparison over the surveyed works.

## 6. Conclusions and Future Work

Navigational assistance for the Visually Impaired (VI) is undergoing a monumental boom thanks to the developments of Computer Vision (CV). However, monocular detectors or depth sensors are generally applied in separate tasks. In this paper, we derive achievability results for these perception tasks by utilizing real-time semantic segmentation. The proposed framework, based on deep neural networks and depth sensory segmentation, not only benefits the essential traversability at both short and long ranges, but also covers the needs of terrain awareness in a unified way.

We present a comprehensive set of experiments and a closed-loop field test to demonstrate that our approach strikes an excellent trade-off between reliability and speed and reaches high effectivity and versatility for navigation assistance in terms of unified environmental perception.

In the future, we aim to continuously improve our navigation assistive approach. Specifically, pixel-wise polarization estimation and multi-modal sensory awareness would be incorporated to robustify the framework against cross-season scenarios. Deep learning-based depth interpolation would be beneficial to enhance the RGB-D perception in high dynamic environments and expand the minimum/maximum detectable range. Intersection-centered scene elements including zebra crosswalks and traffic lights would be covered in the road crossing context. Hazardous curbs and water puddles would be addressed to further enhance traversability-related semantic perception by our advanced version of CNNs using hierarchical dilation. In addition, we are interested in panoramic semantic segmentation, which would be useful and fascinating to provide superior assistive awareness.

Moreover, it is necessary to run a larger study with visually-impaired participants to test this approach, while different sonification methods and audio output settings could be compared in a more general usage scenario with semantics-aware visual localization.

## Figures and Tables

**Table 1:** Layer disposal of our proposed network. “Out-F”: Number of Feature maps at the layer’s Output, “Out-Res”: Output Resolution for input size of 640 × 480 RGB images from the smart glasses, “C”: number of semantic prediction Classes. Encoder: Layers 1–16, decoder: Layers 17–19.

Layer	Type	Out-F	Out-Res
0	Scaling 640 × 480	3	320 × 240
1	Down-sampler block	16	160 × 120
2	Down-sampler block	64	80 × 60
3–7	5 × Non-bt-1D	64	80 × 60
8	Down-sampler block	128	40 × 30
9	Non-bt-1D (dilated 2)	128	40 × 30
10	Non-bt-1D (dilated 4)	128	40 × 30
11	Non-bt-1D (dilated 8)	128	40 × 30
12	Non-bt-1D (dilated 16)	128	40 × 30
13	Non-bt-1D (dilated 2)	128	40 × 30
14	Non-bt-1D (dilated 4)	128	40 × 30
15	Non-bt-1D (dilated 8)	128	40 × 30
16	Non-bt-1D (dilated 2)	128	40 × 30
17a	Original feature map	128	40 × 30
17b	Pooling and convolution	32	40 × 30
17c	Pooling and convolution	32	20 × 15
17d	Pooling and convolution	32	10 × 8
17e	Pooling and convolution	32	5 × 4
17	Up-sampler and concatenation	256	40 × 30
18	Convolution	C	40 × 30
19	Up-sampler	C	640 × 480

**Table 2:** Runtime and accuracy analysis of our architecture compared to other networks that are also designed for real-time applications in the state of the art. IoU: Intersection-over-Union, Fwt: Forward pass time. The runtime are tested on a cost-effective GPU GTX1050Ti.

Architectures	IoU on ADE20K [57]	Fwt at 320 × 240	Fwt at 448 × 256	Fwt at 640 × 480
UNet [62]	28.5%	27 ms	43 ms	131 ms
SegNet [60]	55.6%	N/A	69 ms	178 ms
ENet [61]	58.4%	15 ms	17 ms	**24 ms**
SQNet [63]	55.5%	33 ms	44 ms	89 ms
LinkNet [67]	56.5%	N/A	**14 ms**	32 ms
ERFNet [65]	63.1%	15 ms	20 ms	44 ms
Our ERF-PSPNet	**66.0%**	**13 ms**	16 ms	34 ms

**Table 3:** Accuracy analysis on ADE20K [57].

Networks	Sky	Floor	Road	Grass	Sidewalk	Ground	Person	Car	Water	Stairs	Mean IoU
UNet [62]	75.3%	22.4%	41.4%	52.7%	5.3%	25.3%	16.8%	21.1%	20.4%	4.2%	28.5%
SegNet [60]	91.3%	61.8%	63.0%	62.8%	36.0%	72.3%	31.4%	63.0%	58.4%	15.6%	55.6%
ENet [61]	89.7%	72.4%	69.4%	56.5%	38.2%	75.0%	26.7%	64.8%	67.3%	23.7%	58.4%
SQNet [63]	92.2%	59.1%	66.7%	65.1%	37.0%	68.8%	31.2%	54.2%	63.0%	17.1%	55.5%
LinkNet [67]	91.3%	63.5%	66.3%	63.5%	35.6%	71.6%	30.6%	61.0%	66.6%	15.4%	56.5%
ERFNet [65]	**93.2%**	77.3%	71.1%	64.5%	46.1%	76.3%	**39.7%**	70.1%	67.9%	24.1%	63.1%
Our ERF-PSPNet	93.0%	**78.7%**	**73.8%**	**68.7%**	**51.6%**	**76.8%**	39.4%	**70.4%**	**77.0%**	**30.8%**	**66.0%**

**Table 4:** On the real-world TerrainAwarenessDataset [71] in terms of traversable area parsing. “With Depth”: Only the pixels with valid depth information are evaluated using pixel-wise accuracy.

Approaches	IoU	Pixel-Wise Accuracy	With Depth	Within 2 m	2–3 m	3–5 m	5–10 m
3D-RANSAC-F [9]	50.1%	67.2%	73.3%	53.9%	91.8%	85.2%	61.7%
UNet [62]	52.1%	59.6%	57.1%	56.9%	48.1%	54.0%	56.5%
SegNet [60]	73.1%	90.1%	89.5%	86.1%	84.0%	87.7%	89.3%
ENet [61]	62.4%	85.2%	88.4%	79.9%	84.3%	89.7%	93.1%
LinkNet [67]	74.1%	89.2%	93.8%	93.4%	92.8%	93.5%	93.5%
Our ERF-PSPNet	**82.1%**	**93.1%**	**95.9%**	**96.0%**	**96.3%**	**96.2%**	**96.0%**

**Table 5:** ERF-PSPNet on the real-world dataset [71] in terms of terrain awareness. “Traversability”: Accuracy of the traversable area parsing. Depth information of sky is too sparse to calculate reasonable accuracy values at different ranges.

Accuracy Term	Sky	Traversability	Ground	Sidewalk	Stairs	Water	Person	Car
IoU	88.0%	82.1%	72.7%	55.5%	67.0%	69.1%	66.8%	67.4%
Pixel-wise Accuracy	95.3%	93.1%	81.2%	93.1%	90.1%	86.3%	90.8%	93.1%
With Depth	N/A	95.9%	84.9%	93.1%	90.8%	89.8%	90.4%	92.7%
Within 2 m	N/A	96.0%	76.9%	95.0%	91.9%	96.2%	97.7%	94.3%
2–3 m	N/A	96.3%	81.7%	96.5%	91.9%	82.3%	93.7%	95.2%
3–5 m	N/A	96.2%	87.4%	94.5%	89.4%	76.9%	93.6%	90.8%
5–10 m	N/A	96.0%	86.6%	93.6%	93.1%	84.3%	87.4%	91.4%

**Table 6:** Field test results.

Users	Collisions	Times of Asking for Help	Traversing Time to Reach the Stairs
User 1	1	1	208 s
User 2	1	0	91 s
User 3	0	2	123 s
User 4	1	1	157 s
User 5	0	2	323 s
User 6	1	1	143 s

**Table 7:** The questionnaire.

Users	Male or Female	Feedback in Time?	Comfortable to Wear?	Useful?	Advice for Improvement
User 1	Female	Yes	No	Yes	Provide a detailed tutorial
User 2	Male	Yes	Yes	Yes	Feedback about the curbs
User 3	Male	Yes	Yes	Yes	
User 4	Male	Yes	Yes	Yes	Design a wireless prototype
User 5	Female	Yes	Yes	Yes	
User 6	Male	Yes	No	Yes	

**Table 8:** Maturity analysis according to [8].

Features	Aims	Weights	Scores
F1	Real-Time	9.3	7.83
F2	Wearable	8.6	7.83
F3	Portable	5.7	6.33
F4	Reliable	7.1	7.33
F5	Low-Cost	5.0	-
F6	Friendly	4.3	8.17
F7	Functionalities	2.7	9.33
F8	Simple	2.9	4.83
F9	Robust	2.1	5.67
F10	Wireless	1.4	-
F11	Performance	10.0	7.5
F12	Originality	1.4	9.33
F13	Availability	5.0	10.0
F14	Future	6.4	8.83
Total	Maturity	-	44.08

## Abbreviations

The following abbreviations are used in this manuscript:

TAWS	Terrain Awareness and Warning System
WHO	World Health Organization
CV	Computer Vision
VI	Visually Impaired
RGB-D	RGB-Depth
FPS	Frames Per Second
pRGB-D	polarized RGB-Depth
CNN	Convolutional Neural Network
RANSAC	RANdom SAmpling Consensus
IMU	Inertial Measurement Unit
IR	Infrared
RGB-IR-D	RGB-Infrared-Depth
ToF	Time-of-Flight
RGB-IR	RGB-Infrared
FCN	Fully-Convolutional Network
Non-bt-1D	Non-bottleneck-1D
HSV	Hue Saturation Value
BN	Batch-Normalization
IoU	Intersection-over-Union
TP	True Positives
FP	False Positives
FN	False Negatives
PA	Pixel-wise Accuracy
CCP	Correctly-Classified Pixels
LP	Labeled Pixels
dB	decibel
st	semi-tone
GPS	Global Positioning System
SLAM	Simultaneous Localization And Mapping
USB	Universal Serial Bus

References
